# Surgical Resection of a Calcified Amorphous Tumor of a Mitral Valve Leaflet with Distinct Imaging Features

**DOI:** 10.70352/scrj.cr.26-0235

**Published:** 2026-09-09

**Authors:** Masashi Bungo, Hisashi Uemura, Taichi Sakaguchi

**Affiliations:** Department of Cardiovascular Surgery, Hyogo Medical University, Nishinomiya, Hyogo, Japan

**Keywords:** calcified, amorphous, tumor

## Abstract

**INTRODUCTION:**

A calcified amorphous tumor (CAT) is a rare non-neoplastic intracardiac mass, and its preoperative diagnosis remains challenging because of its overlap with other cardiac lesions.

**CASE PRESENTATION:**

A 68-year-old woman was referred for evaluation of a calcified mass confined to the posterior mitral leaflet. Serial non-contrast CT over 5 years demonstrated progressive enlargement of the lesion together with dynamic internal changes, including loss of central calcification. Given the history of cerebral infarction and potential risk of thromboembolism, surgical resection was performed. The mass and mitral valve were resected, followed by mitral valve replacement. Histopathological examination confirmed the diagnosis of a CAT. The postoperative course was uneventful, and no recurrence was observed during 10 years of follow-up.

**CONCLUSIONS:**

This case represents a rare CAT confined to the posterior mitral leaflet without mitral annular calcification or dialysis dependence. Pathological examination suggested cavitary degeneration corresponding to the serial CT changes. Serial imaging findings may provide valuable clues for preoperative diagnosis and surgical decision-making.

## Abbreviations

CAT
: calcified amorphous tumor
MAC
: mitral annular calcification

## INTRODUCTION

CATs are rare non-neoplastic intracardiac lesions characterized by nodular calcium deposits, chronic inflammatory cells, hyalinization, and degenerated blood components.^1)^ Most mitral valve–derived CATs are associated with extensive MAC. Furthermore, CATs are often misdiagnosed as primary cardiac tumors or infective endocarditis due to shared clinical features such as obstruction or thrombus formation, making preoperative imaging diagnosis challenging.

In this study, we report the surgical resection of a mitral valve leaflet–confined CAT without MAC, with specific imaging findings, that achieved favorable outcomes.

## CASE PRESENTATION

A 68-year-old woman with a history of cerebral infarction at the age of 48 was referred to our hospital for further evaluation of a cardiac mass after a heart murmur (Levine grade III/IV systolic murmur) was detected by a local physician.

**Fig. 1:**
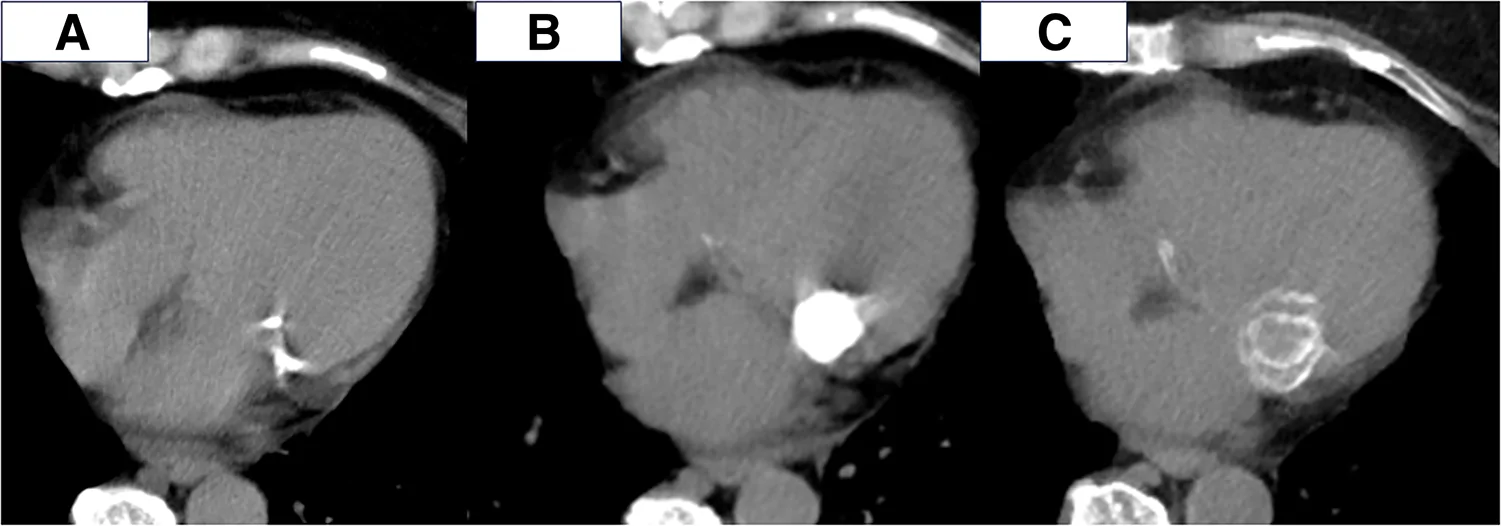
(**A**) Non-contrast CT scan obtained 5 years prior showing localized calcification of the posterior mitral leaflet. (**B**) Non-contrast CT scan obtained 2 years prior demonstrating a mass with central calcification at the posterior leaflet. (**C**) Preoperative non-contrast CT showing loss of central calcification and further enlargement of the lesion.

Transthoracic and transesophageal echocardiography revealed a 20 × 22-mm immobile, calcified mass integrated with the posterior mitral leaflet (**Supplementary Video 1**). She had mild mitral regurgitation with an ejection fraction of 73%. Serial non-contrast CT revealed that a localized calcified lesion of the mitral valve detected 5 years earlier (**Fig. 1A**) had grown into a tumor 3 years later (**Fig. 1B**). Preoperative CT revealed a mass along the posterior leaflet with mixed high- and low-density calcifications (**Fig. 1C**). Laboratory findings were unremarkable. Gradually, the lesion enlarged and showed dynamic alterations in its composition, including loss of central calcification and cavitary changes.

Given the risk of thromboembolism due to the progressive enlargement and history of cerebral infarction, surgical resection of the calcified mass was indicated. A definitive histopathological diagnosis was anticipated after resection.

We performed a median sternotomy and cardiopulmonary bypass, and myocardial protection was achieved using antegrade and retrograde cardioplegia. Right-sided left atriotomy revealed a smooth-surfaced immobile mass extending from the posterior mitral leaflet into the left ventricle. En bloc resection was attempted, but complete sharp dissection was deemed hazardous due to firm adhesion to the posterior left ventricular wall. Instead, the mass was incised and partially resected together with the posterior leaflet. The residual tissue adherent to the ventricular wall was carefully debrided. Approximately 1 mL of a brownish transparent fluid was released from the lesion. Both mitral leaflets were excised, and a 25-mm BICARBON prosthesis (SB; Sorin Biomedica, Saluggia, Italy) was implanted in the intra-annular position.

**Fig. 2:**
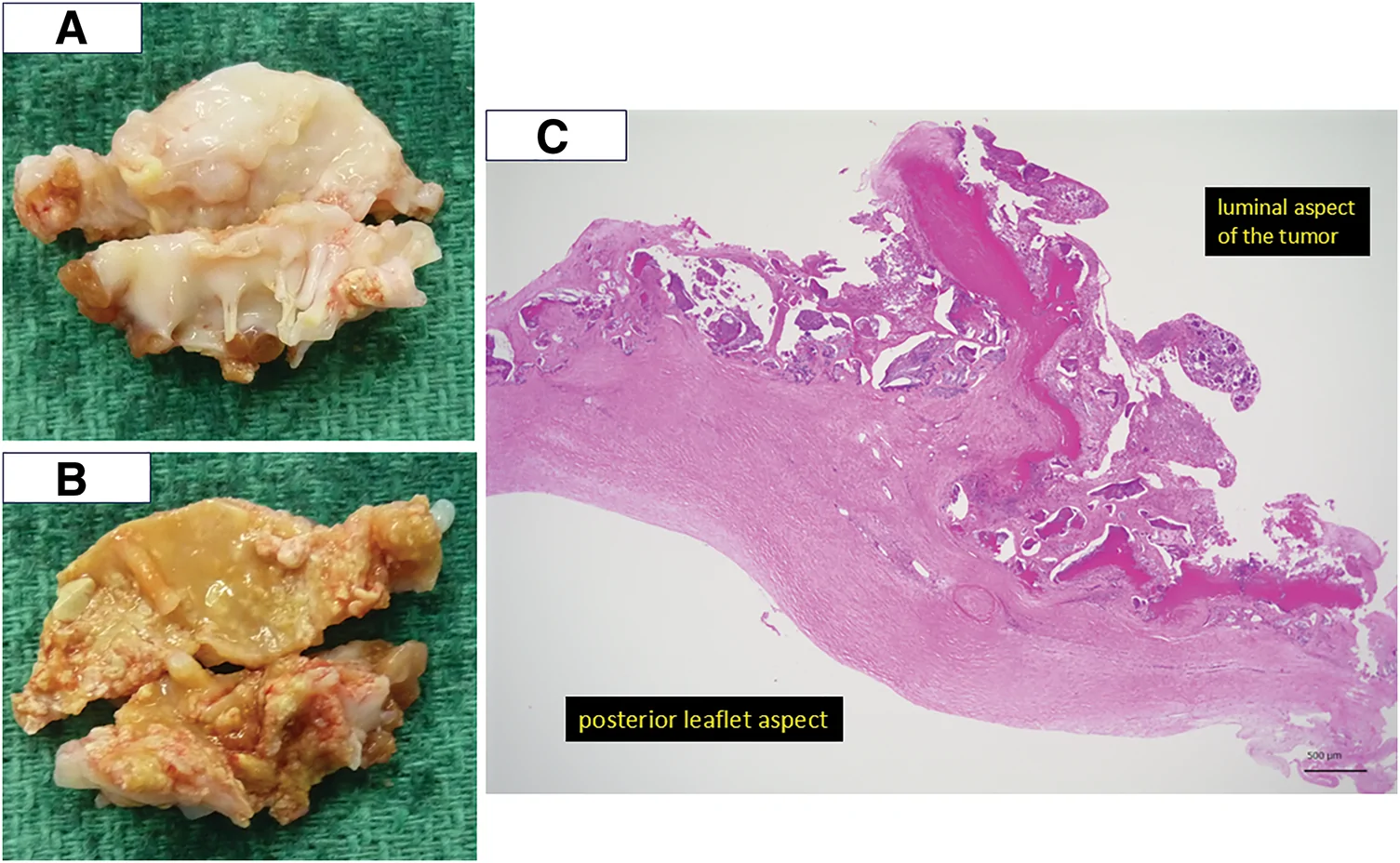
(**A**, **B**) Gross examination showing that the tumor wall was calcified with nodular calcifications scattered within the lumen. (**C**) H&E staining showing numerous island-like calcified nodules, including fibrin, chronic inflammation, and necrotic substances. H&E, hematoxylin and eosin

Macroscopically, the tumor wall was calcified with nodular calcifications scattered within the lumen. Histological examination revealed marked fibrous tissue proliferation and leaflet destruction. No neoplastic cells were detected, regardless of inflammatory cell infiltration (**Fig. 2**). The lesion was diagnosed as a cardiac CAT.

The postoperative course was uneventful, and the patient was discharged on day 17. At the 10-year follow-up, there was no recurrence of the cardiac mass.

## DISCUSSION

CATs, extremely rare non-neoplastic intracardiac masses composed of calcified nodules and amorphous material, are most commonly seen in the mitral valve. However, CATs confined to the mitral leaflet without a MAC are exceedingly rare, and this is the sixth reported case.^2–6)^ Recent studies have shown an association between valvular disease, hemodialysis, MAC, and diabetes mellitus.^7)^ Patients with end-stage renal disease who require dialysis are at a particularly high risk. Nevertheless, our patient had none of these comorbidities, highlighting the unclear etiology and exceptional nature of the presentation.

Accurate imaging is essential for diagnosing and treating cardiac masses. MRI is frequently used, given its superior soft tissue characterization, ability to assess perfusion and vascularity with gadolinium enhancement, and functional assessment. Nonetheless, MRI has limitations, including higher cost, contraindications in patients with implanted devices or severe claustrophobia, longer acquisition times, and inferior sensitivity for calcification compared with CT. CATs typically appear as large, calcified masses extending to the mitral annulus on non-contrast CT scans, whereas on contrast-enhanced scans, they appear as low-density lesions with partial or diffuse calcification, resembling toothpaste.^8)^ They present as polypoid, infiltrative, or broad-based lesions. Differential diagnoses include calcified thrombus, fibroma, myxoma, primary cardiac osteosarcoma, valvular vegetation, and caseous MAC, making preoperative differentiation difficult.

Longitudinal imaging changes may provide additional information for characterizing calcified cardiac masses. Caseous MAC generally occurs in association with extensive MAC, whereas calcified thrombi and cardiac neoplasms may show overlapping imaging features. In the present case, progressive enlargement accompanied by loss of central calcification was a notable finding. However, the specificity of this imaging pattern for CAT and its usefulness in differentiating CAT from other calcified cardiac masses remain uncertain. Therefore, serial CT findings should be considered complementary rather than definitive for diagnosis.

One key finding in the present case was the progressive enlargement of the lesion accompanied by the disappearance of central calcification on serial CT over 5 years. Although the mechanism underlying the disappearance of central calcification remains unclear, several pathophysiological mechanisms may be considered. Calcification loss following cerebral embolism from CAT fragments has been reported,^9)^ and chronic kidney disease and hemodialysis are associated with abnormal calcium-phosphate metabolism and progressive cardiac calcification.^10)^ However, neither condition was present in our patient. Likewise, no evidence of infective endocarditis or other destructive inflammatory processes was identified during observation. Therefore, the imaging changes are more likely to reflect intrinsic remodeling of the lesion rather than systemic metabolic or infectious causes. Progressive fibrin deposition and chronic degenerative remodeling might have contributed to the fragmentation or replacement of the pre-existing calcified core.

Operative/pathological findings demonstrated a cavitary structure containing brownish transparent fluid, necrotic material, fibrin deposition, and surrounding fibrosis. These findings may reflect that the loss of central calcification corresponded to cavitary degeneration attributed to tissue necrosis and liquefactive change rather than simple calcium resorption. The serial CT findings closely correlated with the pathological features, suggesting that the temporal imaging changes reflected the underlying structural evolution of the CAT. This may represent a distinctive imaging feature of CATs.

From a clinical perspective, progressive enlargement and internal structural remodeling may indicate the temporal evolution of CAT. However, the relationship between these imaging changes and embolic risk remains uncertain, and no embolic events occurred during the observation period in the present case. Therefore, serial imaging findings should be interpreted together with other clinical and morphological factors, including lesion size, mobility, symptoms, and patient history, when considering patient management and surgical timing.

## CONCLUSIONS

This case demonstrates that serial CT imaging can reveal dynamic structural remodeling of CAT, including enlargement and disappearance of central calcification, which may reflect underlying cavitary degeneration confirmed pathologically. Recognition of these temporal imaging changes may provide additional information for differential diagnosis and individualized clinical management.

## SUPPLEMENTARY MATERIALS

Video 1 Transesophageal echocardiography revealing a calcified mass integrated with the posterior mitral leaflet.

## DECLARATIONS

### Funding

None.

### Authors’ contributions

M.B. and H.U. conceptualized the study and collected the data.

M.B. wrote the manuscript.

All authors critically reviewed and approved the final version.

### Availability of data and materials

Not applicable. No datasets were generated or analyzed during the current study, as this is a single case report.

### Ethics approval and consent to participate

This work did not require ethical considerations or approval according to our institution’s policy. Informed consent to participate in this study was obtained from the patient.

### Consent for publication

All informed consent for publication was obtained from the patient.

### Competing Interests

The authors declare no competing interests.
